# African Buffalo Movement and Zoonotic Disease Risk across Transfrontier Conservation Areas, Southern Africa

**DOI:** 10.3201/eid2202.140864

**Published:** 2016-02

**Authors:** Alexandre Caron, Daniel Cornelis, Chris Foggin, Markus Hofmeyr, Michel de Garine-Wichatitsky

**Affiliations:** University of Pretoria, Pretoria, South Africa (A. Caron);; CIRAD, Montpellier, France (A. Caron, D. Cornelis, M. de Garine-Wichatitsky);; CIRAD, Harare, Zimbabwe (A. Caron, D. Cornelis, M. de Garine-Wichatitsky);; Victoria Falls Wildlife Trust, Victoria Falls, Zimbabwe (C. Foggin);; South African National Parks, Skukuza, South Africa (M. Hofmeyr);; University of Zimbabwe, Harare (M. de Garine-Wichatitsky)

**Keywords:** African buffalo, Transfrontier Conservation Areas, Africa, bovine tuberculosis, brucellosis, Rift Valley fever, Zimbabwe, South Africa, tuberculosis and other mycobacteria, zoonoses, viruses

## Abstract

We report on the long-distance movements of subadult female buffalo within a Transfrontier Conservation Area in Africa. Our observations confirm that bovine tuberculosis and other diseases can spread between buffalo populations across national parks, community land, and countries, thus posing a risk to animal and human health in surrounding wildlife areas.

Since the early 2000s in southern Africa, Transfrontier Conservation Areas (TFCAs) have been created to promote biodiversity conservation and local development (*1*). Increased connectivity between protected areas is designed to promote wildlife movement, ecosystem functioning, and genetic exchange and lead to increased wildlife populations, which should benefit communities living in these areas (e.g., through tourism and sustainable use of natural resources). Small-scale crop and livestock production are the main livelihood options for poor farmers living in communal lands in TFCAs. The extensive wildlife–livestock–human interface areas in TFCAs potentially result in human–wildlife conflicts, including crop destruction by wildlife, competition for resources between wild and domestic ungulates, livestock predation by wild carnivores, and poaching of wildlife; these conflicts are likely to increase as wildlife populations expand (*2*). The potential for the emergence and spread of infectious diseases is also of concern because of increased contact between wild and domestic hosts (*3*,*4*).

The Great Limpopo TFCA (GLTFCA) was created in 2002 and straddles Mozambique, South Africa, and Zimbabwe. It includes the Limpopo, Kruger, and Gonarezhou National Parks (NPs) and other land-use types surrounding the parks (Figure 1). The African buffalo (*Syncerus caffer caffer*) population in Kruger NP is known to maintain animal diseases, including zoonoses such as bovine tuberculosis (bTB) and brucellosis. Buffalo are also suspected of playing a role in the epidemiology of Rift Valley fever (*5*). In 2009, a bTB strain related to the strain occurring in buffalo in northern Kruger NP was detected in buffalo in Gonarezhou NP, suggesting a recent spread from Kruger NP in South Africa to Gonarezhou NP in Zimbabwe (*6*). Although possible explanations were proposed for this transfrontier spread, including direct transmission from buffalo to buffalo or from an unidentified wild or domestic ungulate species to buffalo (*6*), these modes of transmission were not supported by firm data. We report preliminary results from telemetry studies and visual observations of individually identified African buffalo within the GLTFCA.

**Figure 1:**
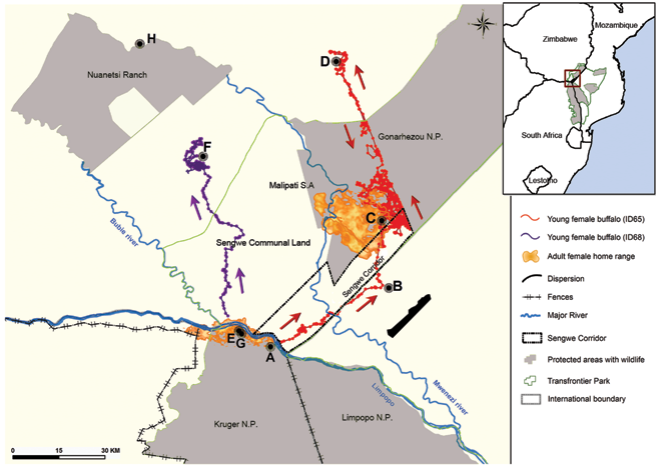
Study area encompassing part of Mozambique, South Africa, and Zimbabwe. Inset map shows location of the Great Limpopo Transfrontier Conservation Area within southern Africa. Orange/yellow shaded areas represent the home ranges of 5 satellite collar–equipped adult female African buffaloes, representative of the 5 herds followed for the study in Kruger National Park (NP) (n = 3) and Gonarezhou NP (n = 2). Because of overlap among the herds, boundaries for the 5 herds cannot be seen. Data for the other adult female buffalo in the study are not represented. The home range of Kruger NP herds span the Limpopo River between South Africa and Zimbabwe. Long-distance movements of 3 subadult female buffalo are shown. Arrows indicate the direction of movements for 2 buffalo; points of capture and resighting are shown for the third buffalo. 1) Red path: a 2.5-year-old female buffalo (ID65) collared at point A in South Africa in October 2013 walked a maximum direct distance of 95 km during January 6–12, 2014. She crossed into Zimbabwe, then into Mozambique (point B), and again into Zimbabwe, where she entered Gonarezhou NP, the home range of a buffalo herd collared during 2008–2010 (point C). She was visually sighted by plane on January 23, 2014, within a 70-strong mixed buffalo herd in the southern part of Gonarezhou NP (H. van der Westhuizen, pers. comm.). On March 14, she left the Gonarezhou NP and entered the Gonakudzingwa cattle commercial ranch area (point D) before coming back into Gonarezhou NP. Inside the park, this buffalo followed a straight line (representing the railway line that crosses the park) and entered Mozambique. 2) Purple path: a 4-year-old young female buffalo (ID68) collared in October 2013 at point E (initially captured but not equipped in July 2011 at age 20 months) walked a direct distance of 64 km. She crossed the Limpopo River on February 26 and, in 8 days, joined the northern tip of the path where a small buffalo herd is thought to range (point F). 3) Path not shown: a 4.5-year-old female, captured at point G in June 2010 at age 24 months, was resighted in March 2013 in an area deep into communal land at a direct distance of 96 km (point H) from capture site. This buffalo was identified on the basis of ear-tag color and number, sex, and estimated age (B. Lessmay, pers. comm.). Movements of all 3 buffalo end outside the Transfrontier Conservation Area (in green).

## The Study

During 2008–2013, a total of 68 satellite or global positioning system radio collars were deployed on African buffalo captured in southern Gonarezhou NP; in northern Kruger NP, south of the Limpopo River; and in Zimbabwe, north of Limpopo River (on Sengwe communal land). Of the 68 buffalo, 47 were adult females, selected because their behavior is representative of core herd movements. Two adult males were also equipped with global positioning system devices because males are believed to move between herds (*7*); however, these devices failed after a few weeks because the collars fell off. Nineteen subadult female buffalo 2.5–4.5 years of age (age determined by teeth eruption) were also selected because individuals from this group are believed to disperse from their native herds (*8*; R. Bengis, pers. comm.). During chemical immobilizations of buffalo, blood samples were taken and stored appropriately for disease screening, and individually numbered ear-tags were applied.

**Figure 2:**
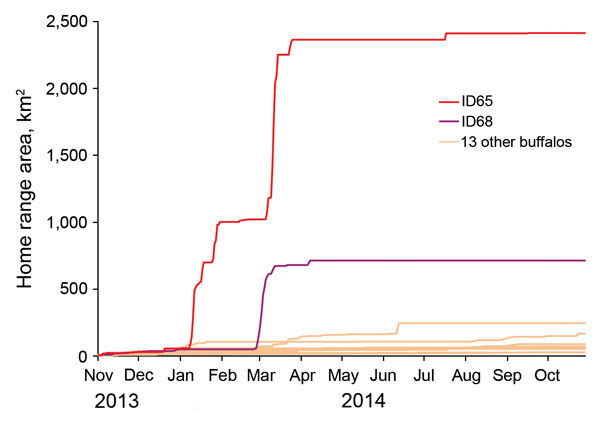
Cumulative home-range area of 15 buffalo collared with global positioning system devices in Kruger National Park, South Africa, in November 2013. Only buffalo with collars that functioned for an entire year are displayed. Data for 2 subadult female buffalo (paths displayed in Figure 1) are shown (ID65 and ID68) separately from data for the 13 other buffalo (subadult and adult females). Gray shading indicates the rainy season (generally when movement began).

During the study, extraordinarily long-distance movements for 3 subadult females were plotted by satellite telemetry readings (Figure 1). In January 2014, a 2.5-year-old female buffalo collared in South Africa walked a maximum direct distance of 95 km. In 6 days, she crossed into Zimbabwe, then into Mozambique, and into Zimbabwe again to enter Gonarezhou NP, with localizations within the home range of the buffalo herd in which bTB was first diagnosed in a female buffalo in 2008 (*6*). This subadult buffalo later left the park and visited a commercial farm area before reentering Gonarezhou NP. In February 2014, another 4-year-old female buffalo walked a direct distance of 64 km in 8 days. Finally, in March 2013, a 4.5-year-old female captured in July 2011 was sighted in a location deep into communal land at a distance of 96 km from her capture site. In contrast to these young female buffalo, no adult females collared in this study moved such long distances outside their home range during 2008–2014. The long-distance travel of these 3 subadult females occurred over a few days during the rainy season (Figure 2) and included movements outside the GLTFCA boundary. 

## Conclusions

Our findings strengthen the hypothesis that bTB was spread from Kruger NP to Gonarezhou NP through buffalo-to-buffalo transmission by subadult females that dispersed from their native herds. Buffalo populations in Kruger and Gonarezhou NPs are connected through long-distance movements of individuals, specifically prebreeding heifers. Although this movement is important for buffalo conservation in TFCAs, it could also facilitate the spread of animal diseases, including zoonoses, across borders. In 2010 and 2011, bTB, Rift Valley fever, and brucellosis were detected in Kruger NP buffaloes, although a previous study failed to detect brucellosis in the Gonarezhou NP population (*9*) (Table). Buffalo ID65, which was initially captured in Kruger NP, was seen among a breeding herd in Gonarezhou NP, indicating the possibility of direct, buffalo-to-buffalo transmission of bTB by dispersing infected individuals, without the need for bridge hosts (e.g., other wild or domestic ungulate species) (*10*).

**Table:** Diagnostic results for bovine tuberculosis, brucellosis, and Rift Valley fever in African buffalo populations sampled in Kruger National Park, South Africa, and on the northern bank of the Limpopo River, Sengwe Communal Land, Zimbabwe, 2010 and 2011*

Type of infection tested for†	No. positive/no. total								
	Calf			Subadult			Adult		All animals‡
	M	F		M	F		M	F	
Bovine tuberculosis	0/4	0/5		2/11	4/25		0/9	2/23	8/77 (4/38)
Brucellosis	0/4	1/5		1/12	4/28		2/9	5/25	13/83 (0/38)§
Rift Valley fever	0/4	0/5		0/12	1/31		1/9	3/28	5/89 (2/38)

*Individuals and herds belong to the same population (see Figure 1, adult female home range). Calf, <2.5 y of age; subadult, 2.5–4.5 y; adult, >4.5 y. †Laboratory tests used: for bovine tuberculosis, Gamma-interferon test and isolation; for brucellosis, Rose Bengal and Complement fixation tests; for Rift Valley fever, indirect ELISA test. ‡Published results (*9*) for the same diseases tested in the Gonarezhou National Park buffalo population in Zimbabwe are shown in parentheses. §Difference between Kruger and Gonarezhou population results for brucellosis was significant by Fisher exact test for equality of proportion for small samples (p<0.02).

Additional ecological information on buffalo dispersion is needed: frequency of dispersion events; size, age, and sex composition of the dispersing groups; and information about whether dispersed individuals later return to their home ranges. Subadult females appear to be particularly prone to dispersing behavior, unlike adult females, and we speculate that they may do so in small groups of individuals that are approximately the same age (*8*). No record exists of subadult female buffalo mixing with male bachelor groups, which are also known to connect to adjacent herds (*7*). So far, the drivers of such movement patterns are unclear. One possible explanation may be an out-breeding mechanism (*11*) that occurs before the start of reproduction; subadult females may leave their native herd to begin their reproduction in a distant herd to minimize in-breeding. Furthermore, abundant resources (i.e., water and grazing areas) available during the rainy season maximize the probability of success of such behavior.

We found that subadult females were infected with bTB, brucellosis, and Rift Valley fever (Table), diseases with different mechanisms of transmission. Age and social position in the herd may influence individuals’ rate of exposure to pathogenic infections and consequently may affect the dynamics of infection within and between herds. Our results indicate that subadult female buffalo could play a role in the spread of diseases among distant populations, across protected areas and international borders, and during the rainy season. This seasonal pattern contrasts with the timing of most wildlife and livestock contact between adult females, which has been observed to occur predominantly during the dry season in the study area (*4*). Buffalo have been observed far outside the boundaries of protected areas, even outside the GLTFCA, in communal land where livestock farming is the main livelihood; these observations considerably widen the wildlife–livestock interface area where disease spread can occur (*12*). Wildlife–livestock interfaces can encompass large areas, rather than being a fence or strip of land at the edge of protected areas. These data should assist in refining disease modeling by showing the importance of temporal and spatial considerations and by redefining variables (e.g., age and sex) involved in risk for pathogen spillover or emergence (i.e., identifying super-spreaders) (*13*).

Our results suggest that the spillover of bTB and other zoonoses at the wildlife–livestock–human interface constitutes a risk to animal and human health in the GLTFCA (*9*,*14*). The health issue in TFCAs cannot be overlooked and must be part of any management decision. Combining ecological and epidemiologic knowledge is necessary to understand disease dynamics in these complex agro-ecosystems.
